# Mid-Term Clinical and Radiological Results at a Tertiary Care Hospital in Türkiye: Is Residual Varus Really Important After Mechanically-Aligned Total Knee Arthroplasty?

**DOI:** 10.7759/cureus.35066

**Published:** 2023-02-16

**Authors:** Abdurrahman Aydin, Sarper Gürsu, Furkan Yapici, Volkan Gür

**Affiliations:** 1 Department of Orthopedics and Traumatology, Düzce Akçakoca State Hospital, Düzce, TUR; 2 Department of Orthopedics and Traumatology, University of Health Sciences, M. S. (Metin Sabancı) Baltalimani Bone and Joint Diseases Research Hospital, Istanbul, TUR; 3 Department of Orthopedics and Traumatology, Erzincan Binali Yıldırım University Faculty of Medicine, Erzincan, TUR

**Keywords:** clinical and radiological results, knee osteoarthritis, total knee arthroplasty, mid-term follow-up, residual varus, mechanical axis, gonarthrosis

## Abstract

Introduction: Traditionally, in total knee arthroplasty (TKA), it is aimed to keep the mechanical axis of the lower extremity neutral (mechanical alignment: 3° varus-valgus in the coronal plane) to improve long-term outcomes. This study aimed to assess the mid-term radiological and clinical results of patients with postoperative residual varus (more than 3° of varus) after mechanically-aligned TKA.

Methods: A total of 616 individuals who had undergone TKA for primary knee osteoarthritis between 2008 and 2013 in our tertiary care hospital were retrospectively examined. All TKAs were performed with the mechanical alignment strategy. For radiological evaluation, hip-knee-ankle (HKA) angle, knee alignment angle (KAA), mechanical medial proximal tibial angle (mMPTA), knee inclination (KI), joint line orientation angle relative to ground (JLOA-G), posterior tibial slope (PS), joint line convergence angle (JLCA) were measured. Besides, patients’ latest radiographs were screened for any clue of aseptic loosening or mechanical failure. Knee Society Score (KSS) (knee and functional subgroups), and Lysholm, Oxford, and Tegner scores were used for clinical evaluation. In addition, knee flexion and extension limitations were assessed.

Results: After applying the exclusion criteria, a minimum of five-year follow-up result of 110 patients was demonstrated. There were 101 females (92%) and nine males (8%). The mean follow-up time was 65.8 ± 6.3 months (range: 60.8-75.8 years). The mean age was 65.9 ± 7.7 years (range: 39 to 89 years). The preoperative mean mechanical axis angle of the lower extremity was 17.3° ± 7.8° (range: 13.4-43.9°), whereas it was 8.3° ± 3.6° (range: 3.2-19.8°) postoperatively. The preoperative mean flexion angle was 90.7° ± 23.8° (range: 40-130°), and the extension limitation was -2.5° ± 7.4° (range: -40-0°), whereas, postoperatively, they were 102.8° ± 15.4° (range from 40° to 150°) and -3.7° ± 7.5° (range from -40° to 0°), respectively. The latest follow-up's mean KSS knee subgroup was 67 ± 18.4 (range: 12-93), the mean KSS functional subgroup was 74 ± 23.6 (range: 20-100), the mean Lysholm score was 81.7 ± 15.7 (range:25-100), the mean Tegner score was 3.65 ± 0.99 (range: 1-5), the mean Oxford score was 37.4 ± 6.5 (range: 9-48). There was no patient with aseptic loosening or mechanical failure.

Conclusions: In the mid-term follow-up of patients with residual varus after mechanically-aligned TKA, satisfactory clinical and radiological results were obtained without aseptic loosening or implant failure.

## Introduction

Until recently, the lower extremity alignment goal after total knee arthroplasty (TKA) was a neutral hip-knee-ankle (HKA) angle, while the goal in knee alignment angle (KAA) was 6-9° valgus [1]. While mechanical alignment is thought to be more beneficial, ideal alignment has become controversial in the current literature. The requirement for each patient's HKA angle to be neutral is now being questioned [2].

Some studies have focused on innovative alignment solutions to increase TKA’s clinical effectiveness [3]. A current concept introduced in these studies investigating clinical efficacy is the constitutional varus concept. The constitutional varus means that the patients' existing lower extremity mechanical axis alignment is more than 3° of varus at the time of completing bone-skeletal maturation [4]. Based on this concept, the kinematic alignment method was introduced. Kinematic alignment (KA) is a technique for restoring the natural kinematic axis of knee joints [5]. The notion of KA might be utilized as a reference throughout TKA in all abnormalities, and the knees could be left in moderate varus to retain the original lower extremity alignment and limit soft tissue release. This strategy might potentially improve patient satisfaction following TKA [6].

Many recent studies have shown that varus alignment has good functional and clinical results, although that was not the case in previous studies [7,8]. The improvements in the implant design, particularly in the construction of polyethylene inserts and components, have significantly reduced wear on shared surfaces and reduced implant problems due to component misalignment. Furthermore, a complete correction of the lower extremities in individuals with preoperative varus caused an abnormal increase in the Q angle and caused severe instability in the knee [9]. Therefore, it was stated that by allowing an adequate residual varus in individuals with varus knee osteoarthritis, TKA could be more successful [6].

This study aimed to investigate the mid-term radiological and clinical outcomes of individuals with postoperative HKA angles of more than 3° of varus (residual varus). We hypothesize that the residual varus after TKA does not lead to aseptic loosening or mechanical failure, and the patients are still satisfied with their condition in the mid-term. Although there are comparative articles examining alignment in the literature, publications that present mid-term results are limited. Previous studies provide limited data regarding both clinical and radiological evaluation [10,11].

## Materials and methods

This study was authorized by the Institutional Review Board of the University of Health Sciences, Metin Sabanci Baltalimani Bone and Joint Diseases Research Hospital, Istanbul, Türkiye (IRB No. 48865165-302.14.01). Individuals having varus-type primary knee osteoarthritis who had TKA between January 1, 2008, and December 31, 2013, were included in the study. Informed consent was obtained from all patients participating in the study. The study was conducted at the University of Health Sciences, Metin Sabanci Baltalimani Bone and Joint Diseases Research Hospital, Istanbul, Türkiye.

**Figure 1:**
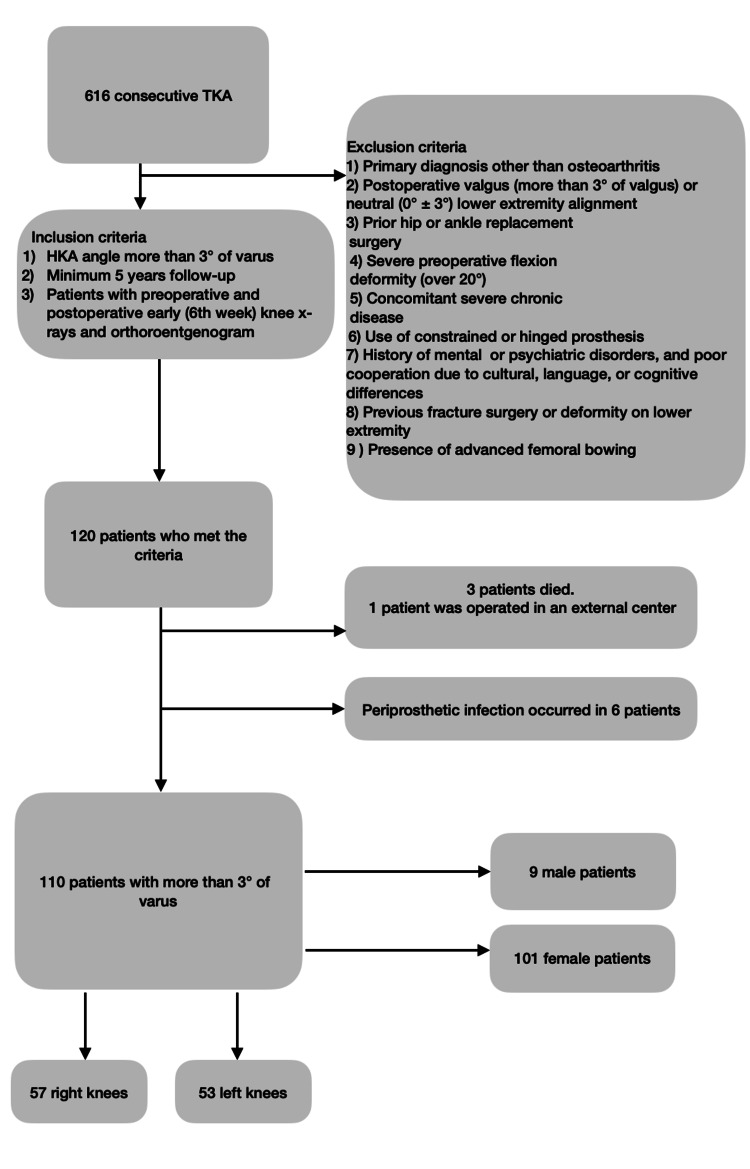
Inclusion and exclusion criteria. HKA, hip knee ankle; TKA, total knee arthroplasty.

After the application of the criteria of inclusion and exclusion, the remaining 110 patients were evaluated (Figure 1). The average age was 65.9± 7.7 years (range 39-89). The average follow-up time was 65.8 ± 6.3 months (range 60.8-75.8).

Surgical management 

Following a conventional midline skin incision, a medial parapatellar technique was utilized. The menisci and the anterior cruciate ligament were entirely removed, and the patella was extended. Osteophytes were removed from the femoral notch. According to preoperative radiological planning, the entrance location of intramedullary guides for the femur was designated ventrodorsally and mediolaterally with a 5° or 6° valgus angle using a gouge. The Whiteside's line (anteroposterior axis) and the anatomical transepicondylar axis were employed as markers for proper rotational orientation of the femoral component, along with the posterior condylar axis. The proximal and distal reference points for the tibia were the center of the tibial intercondylar eminence and the actual center of the ankle, respectively. A cemented complete knee prosthesis was inserted in all TKA patients. Patients were treated with peripatellar osteophytes removed and patellar denervation rather than patellar replacement.

**Figure 2:**
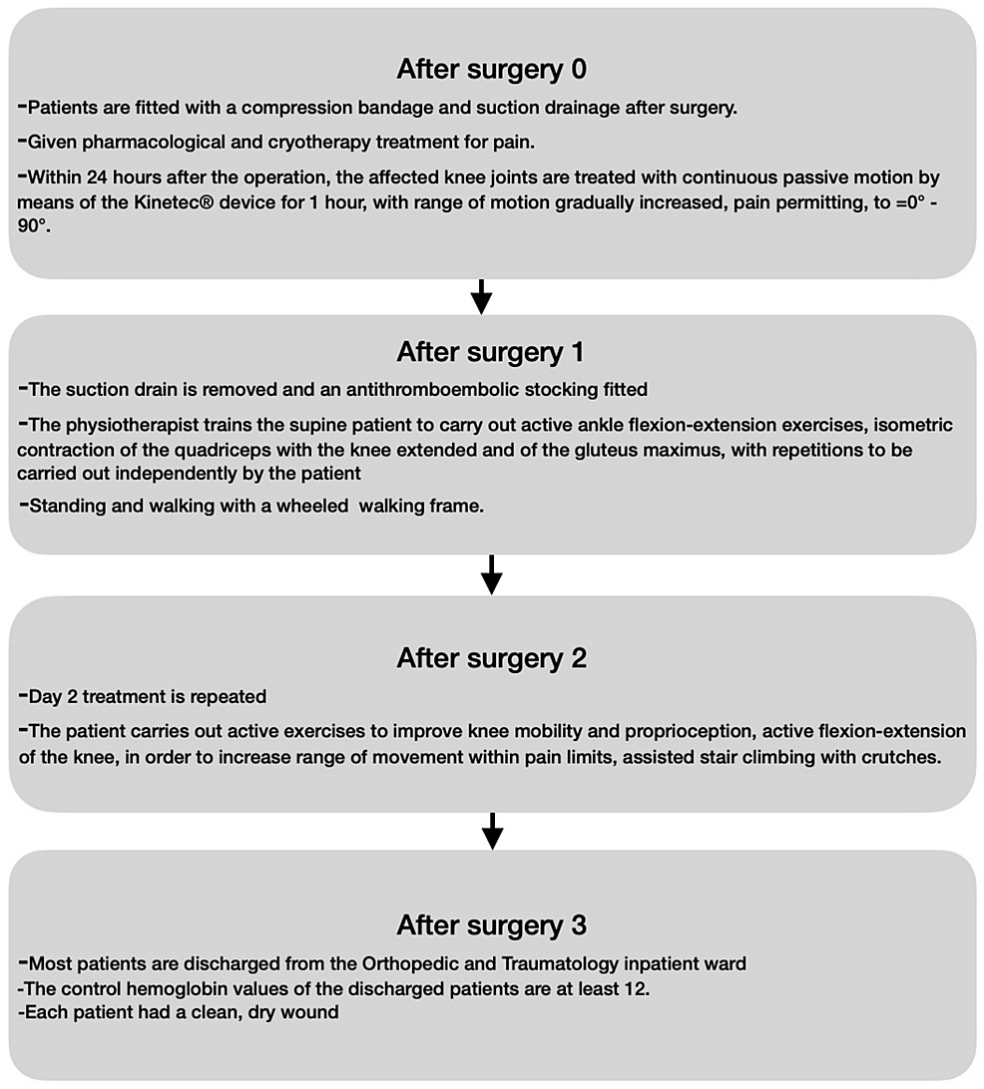
Rehabilitation and discharge protocol.

After TKA and the rehabilitation process, patients were allowed to be discharged from the facility per a standard protocol; before discharge, all participants had a dry and clean wound and could walk with flexion at least 900° [12]. All patients had an independent and objective radiological and clinical evaluation before surgery, after surgery, and at the latest follow-up. The rehabilitation and discharge protocol is given in Figure 2.

Follow-up and observation indices 

A single doctor (AA) filled in the functional score, knee score (Knee Society Score), and the scales of Lysholm, Tegner, and Oxford after TKA and throughout follow-up [13-15]. The knee function and clinical effectiveness were assessed using these scoring methods. In addition, flexion degrees and extension limitations were evaluated at the patients' final follow-up. Complications following surgery were detected and documented.

**Figure 3:**
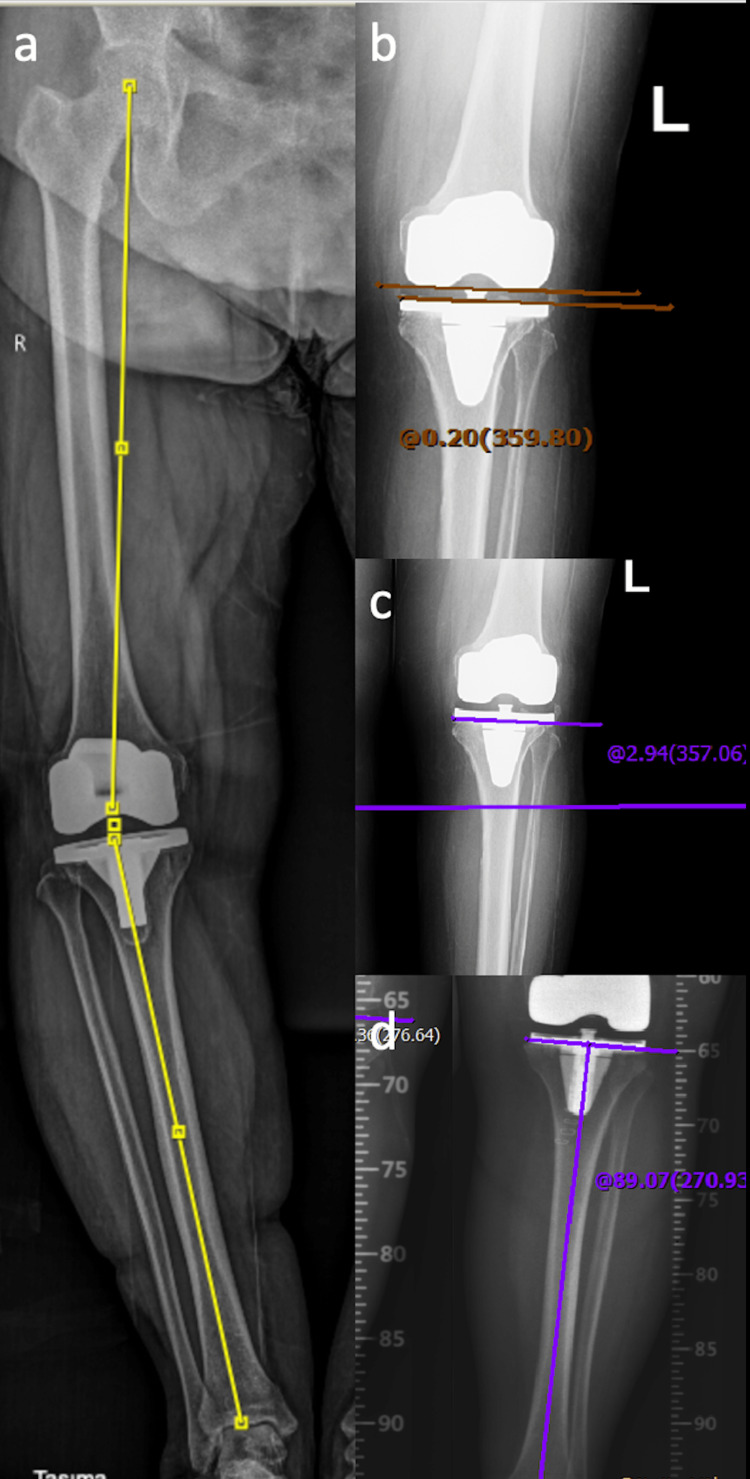
(a) HKA angle measurement, (b) KJLCA measurement, (c) JLOA-G measurement, (d) mMPTA measurement. HKA, hip knee ankle; KJLCA, knee joint line convergence angle; JLOA-G, joint line orientation angle relative to the ground; mMPTA, mechanical medial proximal tibial angle

**Figure 4:**
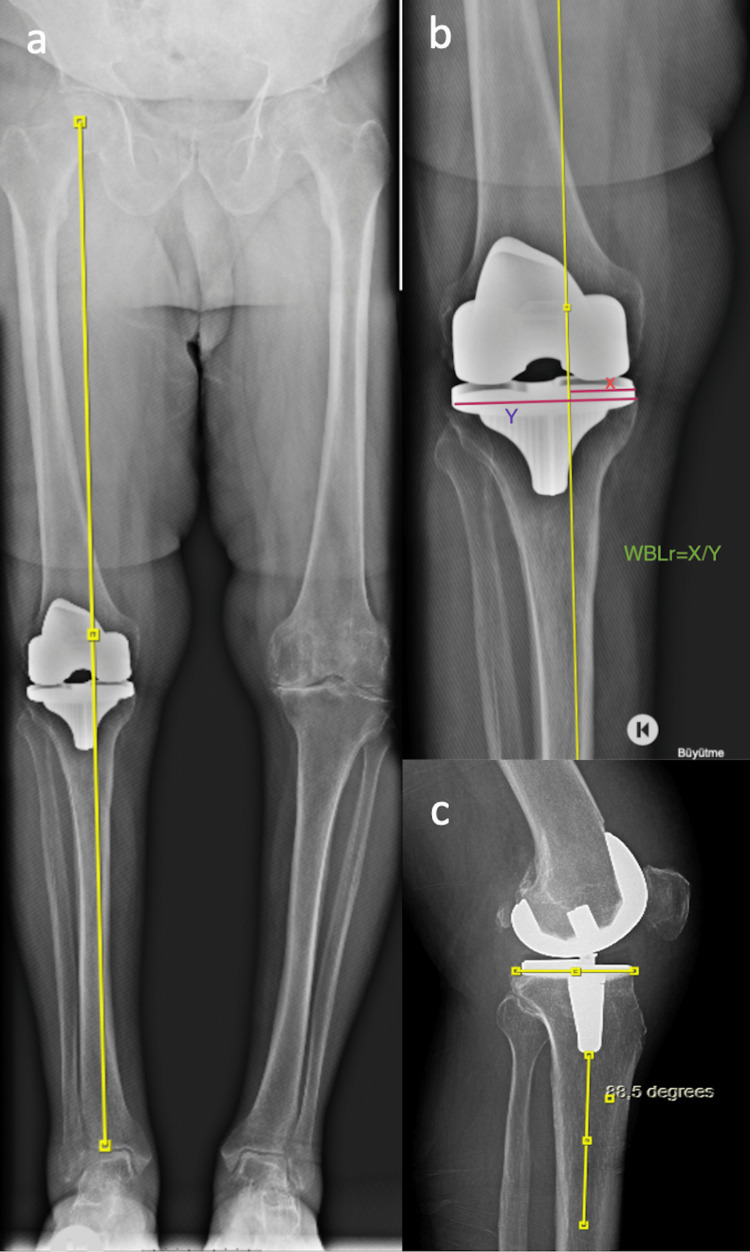
(a) WBL, (b) measurement of WBLr, (c) measurement of posterior tibial slope. WBL, weight-bearing line; WBLr, weight-bearing line ratio

At the final follow-up (65.8 ± 6.3 months, range: 60.8-75.8), full-leg weight-bearing radiographs and lateral/anteroposterior radiographs of the operated knee were obtained. The angle of coronal HKA was denoted as the included angle formed between the mechanical tibial axis and the mechanical femoral axis. The HKA angle was measured as a deviance from 180^o^, with varus alignment being positive and valgus alignment being negative. The weight-bearing line (WBL) is traced from the femoral head's center to the talus's center. WBL ratio is obtained by subdividing the length between the point where the WBL cuts the tibial plateau and the medial tibial plateau edge by the entire tibial plateau width. The measure of the angle created medially between the mechanical axis of the tibia and the proximal tibial joint line is known as mechanical medial proximal tibial angle (mMPTA). Knee inclination (KI)-joint line orientation angle related to the ground (JLOA-G) expresses the angle measurement between the line drawn parallel to the ground and the joint line of the proximal tibia. Knee joint line convergence angle (KJLCA) refers to the angle measurement between the femoral distal joint line (the line is between the endpoints of the femoral condyles) and the tibia proximal joint line (the line joining the concave points of the subchondral lines of the tibial plateaus). The anatomical incline of the tibial plateau to the posterior in the sagittal plane is known as the posterior tibial slope (Figures 3-4). 

The angle formed by the tibia and femur's anatomical axis is named the KAA. Furthermore, the loosening risk was assessed using periprosthetic radiolucent lines.

Statistical analysis 

The data analysis was performed using the IBM SPSS Statistics for Windows, Version 22.0 (Released 2013; IBM Corp., Armonk, New York, United States) and represented as mean standard deviation. Depending on the distribution features of the data, the Mann-Whitney U or the Student's t-tests were used to examine all continuous variables. The Kolmogorov-Smirnov test was used for the normality analysis of the data. It was seen that the data had a normal distribution except for postoperative WBL ratio (WBLr) and preoperative mMPTA. A p-value < 0.05 was regarded as significant in all comparisons.

## Results

Of the 110 patients, 101 were female (92%), and nine were male (8%). The mean age of the participants was 65.9 ± 7.7 (range: 39-89) years. While the mean preoperative HKA angle of the patients was 17.3° ± 7.8° (range: 13.4°-43.9°), the mean postoperative HKA angle was 8.3° ± 3.6° (range: 3.2°-19.8°) (p <.001). The average alteration in HKA was found to be -8.9° ± 7.39° (range: 0.6° -30.1°). Although the average postoperative HKA angle was in varus, the negative change indicates a valgus change.

The data of the patients whose radiological parameters were evaluated before and after surgery were given in Table 1. There were statistically significant differences in WBLr, KAA, JLOA-G, mMPTA, and JLCA in patients before and after surgery. When the angular measurements were compared before and after surgery, it was seen that the change in radiological parameter measurements, except for the JLOA-G angle change, was above 5 degrees. Although the measurement in JLOA-G angle was statistically significant before and after surgery, there was a 1.2° difference between postoperative and preoperative measurements.

**Table 1:** Comparison of radiological parameters before and after surgery WBLr, weight-bearing line ratio; KAA, knee alignment angle; JLOA-G, joint line orientation angle relative to the ground; mMPTA, mechanical medial proximal tibial angle; JLCA, joint line convergence angle

	Before/After Surgery	N	Mean	Std. Deviation	Min-Max	P value
WBLr	Preoperatively	110	-0.07	0.27	-0.6-1.56	P<0.001
	Postoperatively	110	0.24	0.1	-0.25-0.55	
	Mean value of change	110	0.31	0.39	-0.95-0.86	
KAA	Preoperatively	110	9.7	7,6	13.4-43.9	P<0.001
	Postoperatively	110	1	3,3	5.8-12.3	
	Mean value of change	110	8.7	7.4	19.5-29.3	
JLOA-G	Preoperatively	110	4.9	3.08	-3.6-20.6	P<0.001
	Postoperatively	110	3.7	2.9	3.5-13.5	
	Mean value of change	110	1.7	1.4	-8.1-23.5	
MPTA	Preoperatively	110	81.1	7.7	71.7-90.1	P<0.001
	Postoperatively	110	86.1	2.6	74.4-91.8	
	Mean value of change	110	5.4	9.1	6.9-19.7	
JLCA	Preoperatively	110	6.79	4.68	5.4-36.6	P=0.04
	Postoperatively	110	1.06	1.75	1.7-9.9	
	Mean value of change	110	5.7	4.8	5.4-36.6	

In the clinical evaluation of the patients preoperatively, the knee flexion angle was 90.7° ± 23.8° (range: 40°-130°), and the extension limitation was -2.5° ± 7.4° (range: -40°-0°). At the latest follow-up, the knee flexion angle was 102.89° ± 15.43° (range: 50°-160°), and the extension limitation was -3.72° ± 7.52° (range: -50°-0°). There was no evidence of prosthetic loosening or mechanical failure. Six patients with periprosthetic joint infections were excluded according to the exclusion criteria. Throughout the follow-up period, no knees needed reconstructive surgery. The last follow-up KSS and Lysholm, Tegner, and Oxford scores were satisfactory. The clinical scores evaluated during the latest follow-up were demonstrated in Table 2. All patients were pain-free at the latest follow-up.

**Table 2:** Evaluation of clinical and functional results at the last follow-up. KSS, Knee Society Score KSS: 80-100-excellent, 60-80 good, 40-60 fair, below 40 poor; Lysholm score: 91-100 excellent, 84-90 good, 65-83 fair, below 64 unsatisfactory; Tegner score: 4 work moderately heavy labor (e.g., truck driving, etc.), 3 work-light labor (nursing, etc.); Oxford score: 40-48 satisfactory joint function (may not require any formal treatment), 30-39 mild to moderate knee pain (may benefit from non-surgical treatment, such as exercise, weight loss, and /or anti-inflammatory medication), 20-29 may indicate moderate to severe knee pain, 10-19 may indicate severe knee pain.

	N	Mean	Std. Deviation	Min-Max	Range
Flexion angle	110	102.89°	15.43°	50°-160°	0°-180°
Extension limitation	110	-3.72°	7.52°	-50°-0°	0°-180°
KSS knee score	110	67.04	18.45	12-93	0-100
KSS functional score	110	74.08	23.68	20-100	0-100
Lysholm score	110	81.77	15.78	25-100	0-100
Tegner score	110	3.65	0.99	1-5	0-10

Thirty-eight (34.5%) patients received the implants of TST Medical Devices (Istanbul, Türkiye), 26 (23.6%) patients received implants of Johnson & Johnson (New Brunswick, New Jersey, United States), five (4.5%) patients received implants of Smith & Nephew plc (London, United Kingdom), 24 (21.8%) patients received of Wright Medical Group, Inc. (Memphis, Tennessee, United States) and 17 (15.4%) patients received implants of Zimmer Biomet (Warsaw, Indiana, USA). Posterior Cruciate-retaining and Posterior Cruciate-substituting implants were used in 94 (85.5%) and 16 (14.5%) patients, respectively.

The cements used to fix the components to the femur and tibia were antibiotic (gentamycin) cements produced by each company for their implant.

## Discussion

The study's most important findings were that the functional scores of the patients with residual varus were satisfactory, the latest range of motion of the patients was more than 100^o^, they did not have knee pain, and no implant loosening or implant failure was detected.

For TKA surgery, the most contentious debate is on the question of what optimal alignment should be achieved after surgery [16]. As is common knowledge, attaining the neutral mechanical axis was thought to be a crucial component in the success and durability of knee arthroplasty [17]. It was thought that implant durability increased and patient function improved with the neutral mechanical axis (mechanical alignment) technique applied for primary TKA. Because this technique provided good radiographical alignment and contained fewer axis deviations (axis outliers) [18]. Besides, due to an unbalance of pressures between the lateral and medial tibial plateaus, a higher risk of component loosening and rapid wear of the polyethylene insert may arise from malalignment in the lower extremity coronal plane. Malalignment of the prosthesis was shown to be a major cause for revisions, as demonstrated by several research findings [19]. According to certain studies, deviations of 3° or more from neutral alignment are associated with poor clinical outcomes (especially in those with a varus mechanical alignment) and a decreased probability of long-term survival [20-22]. In addition, a non-neutral alignment was connected to increased aseptic subsidence and loosening, which resulted in revisions [23,24].

Contrary to the studies mentioned above, mild malalignment in current prostheses did not increase the risk of revision, according to several studies [6,20,25-29]. Individuals with a neutral alignment and those with a varus malalignment after surgery had similar clinical outcomes [10]. In addition, a study of the radiological and clinical outcomes of 280 patients who had TKA revealed no significant difference between implant location within the 3^o^ varus-valgus range and implant placement outside these values related to Kaplan-Meier 15-year joint survival expectation [25]. In our minimum five-year follow-up research, we observed that the mean HKA angle decreased from 17.3° ± 7.8° postoperatively to 8.3° ± 3.6 among individuals having deviations of >3° in the lower extremity alignment, indicating continuity in the varus alignment, and a life without complaints. Furthermore, no complications were identified, such as component loosening or failure. This outcome is in line with recent research [6,28,30]. 

We focused on the alignment strategies to expand the above discussion a little further. There are two main alignment approaches in literature: fixed and individualized. Fixed alignment approaches are divided into two categories: mechanical alignment and anatomical alignment, with the goal of achieving consistent objectives for all knees. Individualized alignment strategies include four distinct approaches: adjusted mechanical alignment, unrestricted and restricted kinematic alignments, and functional alignment, aiming for native soft-tissue tension and prioritizing constitutional alignment. Among these approaches, there are four of them commonly used and accepted in literature for the varus knee phenotype: functional alignment, unrestricted kinematic alignment, mechanical alignment, and adjusted mechanical alignment [31].

Functional alignment seeks to achieve soft-tissue balance by equalizing gap heights and reducing variations in joint line obliquity (JLO). The functional alignment strategy consists of applying restricted limits (HKA -6° to 3°, MPTA & lateral distal femoral angle (LDFA) 84° to 93°) and adjusting the final position of the components based on pre-resection soft tissue laxities. Functional alignment requires robotic help for implant placement prior to resection. It does not need soft-tissue release. Functional alignment accomplishes gap balance while preserving JLO.

Unrestricted kinematic alignment seeks to restore constitutional soft-tissue laxities with native JLO. Unrestricted kinematic alignment has a personally customized alignment technique consisting of parallel resections to the native LDFA, MPTA, and posterior condylar axis. Unrestricted kinematic alignment requires caliper-based manual cutting guides, and it is unlikely to cause soft-tissue release, but the coronal and sagittal imbalance is probable. Additionally, there is a possibility of excessive alignments, primarily due to low-precision instruments and cutting guides.

Mechanical alignment is the most prevalent alignment approach for TKA and favors symmetrical compartment loading for prosthetic durability. The mechanical alignment technique comprises neutral JLO and HKA angles. For resection, mechanical alignment utilizes manual cutting guides. It necessitates a significant soft-tissue release; hence, lateral condylar lift-off is probable. Mechanical alignment elevates the medial joint line, elongates the lateral femoral column and limb, and has a greater likelihood of necessitating increased constraint.

Adjusted mechanical alignment aims to position the TKA's components from a neutral starting point toward constitutional alignment. Adjusted mechanical alignment employs an individualized, restricted strategy (HKA -3° to 3°, MPTA & LDFA 83° to 93°) and is the most conservative option. It requires computer or robotic assistance. Adjusted mechanical alignment necessitates medial collateral ligament soft-tissue release, whereas native HKA remains substantially outside the -3* window. Adjusted mechanical alignment does not restore JLO.

According to the findings of some clinical studies, there is no connection between the longevity of components and the alignment of the lower extremities after surgery [8,25,26]. In a retrospective examination of the 15-year survival of 398 knees, neutral alignment did not increase prosthesis lifespan; hence, it was determined that neutral alignment had no substantial influence on modern prosthetic survival prognosis [25]. Recent studies using long-leg radiography to define the true alignment more accurately have found no difference in long-term prosthetic survivorship with implants inadvertently positioned outside the 3° mechanical alignment target, despite long-term multinational registry data of 82% survivorship at 25 years supporting the durability of the mechanical alignment strategy [24,32-35]. It has been observed that at the end of the process of skeletal development, a portion of the general population does not have a neutral mechanical alignment, and it is believed that a part of the general population has some degree of varus in their alignment. If this is the case, obtaining neutral alignment after surgery is aberrant and unnatural in people living with a particular alignment from skeletal development [36]. Natural alignment has been advocated and recommended as a goal alignment for patients in the research by Bellemans [4]. In our study, most patients with postoperative residual varus also had preoperative varus. Based on this, the clinical well-being of the patients in our study can be explained.

Unfortunately, this research had some limitations. Our findings may be influenced by the comparatively short follow-up period (mean 5.6 years). The absence of a control group is another major limitation of the present study. The study should have been better with three comparative groups: valgus malalignment, neutral alignment, and varus malalignment. The lack of functional scores before surgery, despite the complete radiological data of the patients, is another limitation. Verifying component loosening or implant failure due to lower extremity malalignment in participants of this research may require a longer time. Our study population is skewed toward the female population. The prostheses used were not of a single type or brand of implants. Different implants of many brands were used in this study. Due to these limitations, the generalization of the study may be less valuable than expected. The glaring side of the study was the high number of patients.

## Conclusions

The ideal alignment after TKA is a long-standing debate and the subject of many studies. There will be many more studies on this subject.

The results obtained in this study showed that the radiological and clinical outcomes of patients with residual varus were satisfactory with a minimum five-year follow-up. Although there was a severe change in varus deformity after TKA surgery, the patients whose varus alignment continued were still satisfied. Also, the remaining varus deformity after TKA did not lead to component loosening or failure in the mid-term follow-up.
